# Geographical characteristics and other factors associated with alcohol-related fatal fires in Ireland 2014 – 2021

**DOI:** 10.12688/hrbopenres.14341.1

**Published:** 2026-02-10

**Authors:** Anne Doyle, Abigail K. Stevely, Suzi Lyons, James McBride, Fiona Riordan, John Holmes

**Affiliations:** 1 Population Health, School of Medicine and Population Health, University of Sheffield, Regent Court, 30 Regent Street, Sheffield,, The University of Sheffield School of Health and Related Research, Sheffield, England, S1 4DA, UK, UK; 2 Evidence, Health Research Board, Dublin, D02 H638, Ireland; 3 National Directorate for Fire and Emergency Management, Department of Housing, Local Government and Heritage, Dublin, D01 W6X0, Ireland

**Keywords:** Fire, alcohol-related mortality, socioeconomic deprivation, urban-rural location

## Abstract

**Introduction:**

Alcohol-related fatal fires represent a tragic but preventable death. Geographic features of these events can inform prevention campaigns and are important to consider in combination with other risk factors. This study aims to examine the factors associated with alcohol-related versus non-alcohol-related fatal fires, and to examine geographical characteristics of alcohol-related fatal fires in Ireland.

**Method:**

Using Irish Coronial data, we looked at all 273 fire fatalities for the period 2014 to 2021, of which 112 (41.0%) had positive alcohol toxicology. Descriptive analyses were conducted followed by logistic regression and geospatial analyses to understand the characteristics of alcohol-related fatal fires.

**Results:**

Compared to non-alcohol related fatal fires, the proportion of fatal fires that involved alcohol was higher among 35–49-year-olds (65.9%) smokers (54.7%) and those accompanied by friends (86.7%). In regression analyses, history of alcohol dependency was the only significant risk factor for a fatal fire being alcohol-related. Rural areas were over-represented in alcohol-related fatal fires, with an annual average 0.37 deaths per 100,000 people in rural areas for every 0.25 in urban areas. Alcohol-related fires that occurred in rural areas involved a longer travel time and distance to the nearest fire station but there was no significant association between alcohol-related fires and area-level deprivation.

**Conclusions:**

People with a history of alcohol dependency suffer higher rates of alcohol-related vs non-alcohol-related fatal fires. These deaths are avoidable, and it is essential that alcohol use is acknowledged as a contributory risk factor and efforts be made to raise awareness and target at-risk individuals.

## 1. Introduction

Alcohol use is the tenth leading risk factor for both deaths and disability-adjusted life years globally. ^1^ Over 5% of all deaths annually are attributable to alcohol and its use is associated with alcohol dependence and alcohol-related liver disease, along with health conditions including cardiovascular disease and cancer. ^2^ As well as health conditions, alcohol use is commonly linked with traumatic incidents such as road traffic collisions, suicides, drownings, and homicide. ^2– 5^ Alcohol is also a risk factor for residential fire fatalities and existing global evidence indicates that up to one-half of those who died in fire incidents had been drinking prior to the event. ^6– 15^ Fortunately, despite more than 20,000 emergency fire service callouts annually in Ireland, fatalities are relatively rare. ^16^

Although declining, alcohol use remains high in Ireland at 9.9 litres of pure alcohol per adult (aged 15 years or greater) in 2023. ^2^ Of potential concern is the notable shift to drinking at home as opposed to drinking in licensed premises, a trend that has been especially pronounced since the COVID-19 pandemic and exacerbated by the cost-of-living crisis in Ireland. This pattern is also reflected elsewhere. ^17– 21^ Drinking at home, and especially solitary drinking, is linked to heavier drinking and a higher risk of alcohol-related problems. ^22, 23^ High blood alcohol concentration (BAC) levels are likely to reduce mobility, coordination, and sensory responses, affecting the ability to respond to escape or potentially extinguish a fire. ^6, 24^

Alcohol can leave a person more vulnerable to residential fire incidents as otherwise normal behaviours may become hazardous while intoxicated, such as cooking or smoking and then falling asleep, as well disorientation and drowsiness resulting in a slower response or reduced awareness of smoke or an alarm sounding. ^25– 27^ In addition to behavioural risk factors, previous research examining fatal fire incidents has indicated that geographic, individual, and building characteristics, are associated with higher likelihood of residential fire mortality. ^7, 9– 12, 28– 36^

Fatal fires involving alcohol have not been examined in detail in Ireland nor have the geographical characteristics of such events. Studies in other jurisdictions have examined alcohol-related fire fatalities ^6, 10, 31^ but the use of coronial data for such is less common. ^37, 38^ Considering the high level of alcohol use in Ireland, the growing shift to drinking at home, and the prevalence of alcohol involved in fatal fires in Ireland, this exploratory study sought to examine the factors associated with alcohol-related fatal fires, including in comparison to non-alcohol-related fatal fires. It uses geocoded data to conduct a spatial analysis of the location of fatal fires, examining urban-rural location, area of deprivation, the distance and travel time to the nearest fire station. The findings will support fire prevention initiatives, in particular to inform campaigns to reduce alcohol-related fires.

## 2. Methods

### 2.1 Data source and population

Details of all fatal fires that occur in Ireland which are reported to the coroner are collected through the National Drug-Related Deaths Index (NDRDI), regardless of whether alcohol or other drugs are involved. The NDRDI is managed by the Health Research Board (HRB) in Ireland where a team of HRB medical researchers collect data on all fire-related fatalities from multiple sources of data contained in coroners’ files, including postmortem findings, toxicology results, witness testimonials, forensic reports, medical records, and incident reports completed by An Garda Síochána (Irish police service) and/or fires services. Further details on the NDRDI methodology can be found elsewhere. ^39^

All closed inquests into fire fatalities in Ireland during the period 2014 – 2021 were included in this study (n=273), of which 112 (41.0%) had positive alcohol toxicology. For each fire fatality, socio-demographic details were collected and where available, other potential risk factors including history of drug and/or alcohol dependency or misuse, history of mental ill-health, and details about the cause of death. Information on the circumstances surrounding the events of the fatal fire and potential contributory risk factors are recorded, including the location and where it started, the time of the fire, pre-fire behaviour, cause of fire, whether others were present, smoking status, mobility status, and if fire safety equipment (smoke alarms/detectors, fire extinguishers etc.) was in place.

To examine the geographical characteristics of alcohol-related fatal fires, the Eircode (a unique address identifier) of the location of each fire was mapped using Google maps. The rate per 100,000 of the population of each county was then calculated, and using the Pobal HP deprivation Index, the location the fatal fire occurred was assigned a deprivation score. The Pobal HP Deprivation Index, developed by Haase and Pratschke in 2017, uses Census data to determine relative scores of disadvantage or affluence for Ireland’s 18,488 Small Areas. ^40^ Small Areas correspond to between 80 and 120 dwellings. A score is given to the area based on a national average of 0 and ranging from −35 (being the most deprived) to +35 (being the most affluent). For the purposes of this study, these data are presented in eight categories from most deprived to most affluent.

All supplementary tables associated with this study are available in Open Science Framework (OSF) repository at the following link: https://osf.io/hqbzr. ^58^

### 2.2 Statistical analysis

To examine the prevalence of alcohol involvement in fatal fires, we first present descriptive analyses of toxicology results detailing the BAC levels and presence of other drugs detected. A geospatial analysis was completed using Google maps to explore the geographical characteristics of alcohol-related fatal fires, including the urban-rural location, the deprivation score of the location, the travel distance and time to the nearest fire station. The rate of alcohol-related fatal fires per 100,000 of the population of each county was also calculated (and of the urban-rural population of each county) (Supplementary Table 2).

The characteristics of alcohol-related fatal fires for the period 2014 – 2021 were then compared with the characteristics of fatal fires where alcohol was not involved using cross–tabulation and statistical significance was assessed using Pearson *χ* ^2^ tests. Statistical significance was evaluated at a p<0.05.

Due to the modest sample size of fatal fires, only variables identified as being statistically significant in the bivariate analysis were then selected to include in a multivariate logistic regression model as predictors of whether fatal fires were alcohol related. We report the estimated odds ratios (ORs) and 95% confidence intervals (CI). For the regression analyses, a p-value of less than 0.05 was considered to indicate statistical significance. Data analysis was conducted using SPSS version 29.0.1.1 (IBM Corp. Released 2023. IBM SPSS Statistics for Windows, Version 29.0.2.0 Armonk, NY: IBM Corp).

## 3. Results

### 3.1 Alcohol-related fatal fires

During the study period, 2014–2021 inclusive, 273 people died in Ireland due to a fire incident who were reported to the coroner and recorded by the NDRDI. Alcohol was detected on the antemortem or postmortem examination of 112 people, representing 41.0% of all fatal fires. Based on Census population estimates for Ireland, the mean annual fatal alcohol-related fire mortality rate was 0.29 per 100,000 population, ranging from 0.54 in 2014 to 0.08 in 2021. ^41^

**3.1.1 Blood alcohol concentration**

The BAC levels detected in the toxicology results ranged from ‘present’ or ‘trace’, to levels indicating severe intoxication ( Table 1). Of all fatal fires, over one in ten individuals had a BAC reading of 250 mg/100 mL or higher (11.7%) or over one-quarter of alcohol-related fire fatalities (28.6%).

**Table 1.** Blood alcohol concentration (BAC) recorded on toxicology of fatal fires, mg per 100 mL.

Blood alcohol concentration	Number of fatal fires	Proportion of alcohol-related fatal fires (%)	Proportion of all fatal fires (%)
Alcohol not present	161	n/a	59.0
Present/trace (<10 mL)	11	9.8	4.0
10 – 49 mg/100 mL	6	5.4	2.2
50 – 99 mg/100 mL	7	6.3	2.6
100 – 149 mg/100 mL	12	10.7	4.4
150 – 199 mg/100 mL	26	23.2	9.5
200 – 249 mg/100 mL	18	16.1	6.6
250+ mg/100 mL	32	28.6	11.7

**3.1.2 Toxicology findings**

Alcohol was the only drug detected on 47.8% of alcohol-related fires’ toxicology results. A further 14.3% had one other substance detected, and 38.4% had 2+ other substances detected. The most common additional substances detected were antidepressants and benzodiazepines ( Table 2).

**Table 2.** Substances detected on toxicology of those with positive blood alcohol concentration (BAC) toxicology (n=112).

Substance detected on toxicology	Number of alcohol-related fatal fires	Proportion of alcohol-related fatal fires (%)
Alcohol	112	100.0
Antidepressants	31	27.7
Benzodiazepines	31	27.7
Other medication	25	22.3
Other hypnotic or sedatives including Z-drugs	13	11.6
Anticonvulsant drugs	11	9.8
Codeine	5	4.5
Other - 26 substances	26	23.2

### 3.2 Geographical characteristics of alcohol-related fatal fires

**3.2.1 Urban-rural variation of alcohol-related fatal fires**

**Figure 1.**
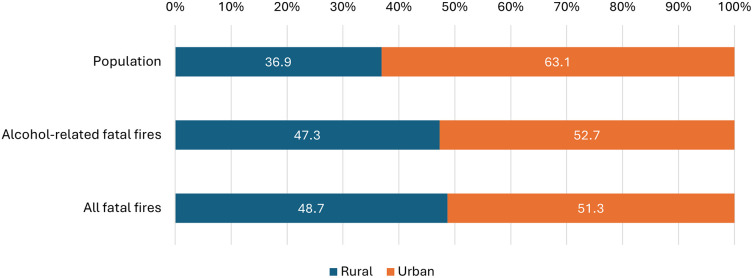
Urban-rural distribution of the Irish population and the location of alcohol-related fatal fires.

Rural areas were over-represented in fatal fires, and this also applied to those that were alcohol-related. According to the Irish Census, an average of 36.9% of the population resided in rural areas during the study period yet rural areas accounted for 47.3% of all alcohol-related fatal fires. ^41, 42^ Urban areas accounted for 63.1% of the population and 52.7% of alcohol-related fatal fires ( Figure 1 and Supplementary Table 3).

**3.2.2 Area-based socioeconomic status of alcohol-related fatal fires**

**Figure 2.**
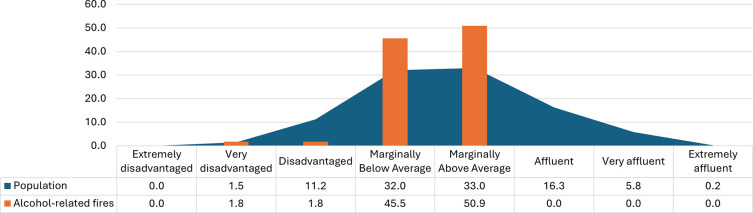
Rate of alcohol-related fatal fires by deprivation octile.

Figure 2 shows the percentage of the population, and that of alcohol-related fire mortalities, by socioeconomic deprivation classification, according to the HP Pobal deprivation index. ^40^ During the study period, 44.7% of the population lived in disadvantaged or marginally below average areas and 49.1% of alcohol-related fatal fires occurred in these areas. ^43^ Although two in every five of the Irish population live in affluent areas (affluent, very affluent, or extremely affluent), no alcohol-related fatal fires occurred in these areas.

**3.2.3 Distance and travel time from nearest fire station to alcohol-related fatal fire location**

**Distance from nearest fire station to alcohol-related fatal fire location**

The mean distance from the nearest emergency response fire station to the location of the alcohol-related fatal fire was seven kilometres (ranging from 200 meters to 28 kilometres). Alcohol-related fatal fires that occurred in rural areas were significantly more likely to be greater distances from the nearest fire station. Over three-quarters of rural alcohol-related fatal fires were five kilometres or greater from the nearest fire station (77.4%) whereas urban alcohol-related fatal fires were more likely to be less than five kilometres from the nearest fire station (64.4%) (p<0.001) (Supplementary Table 4). There was no significant association between distance and level of deprivation of the location the fatal fire occurred (p=0.254) (Supplementary Table 5).

**Travel time from nearest fire station to alcohol-related fatal fire location**

The mean travel time from the nearest fire station to the location of the alcohol-related fatal fire was 10.5 minutes; the quickest journey time was 2 minutes, the longest was 35 minutes. There was no significant association between travel time and area of deprivation (p=0.690). Predictably, urban alcohol-related fatal fires had considerably less travel time from the nearest fire station (less than 5 minutes) (62.7%) compared to rural alcohol-related fatal fires (37.3%) (p<0.001) (Supplementary Table 4). There was no significant association between travel time and level of deprivation of the location the fatal fire occurred (p=0.180) (Supplementary Table 5).

### 3.3 Factors associated with alcohol-related fatal fires

**3.3.1 Characteristics of alcohol-related fire fatalities**

An examination of the characteristics of alcohol-related fire fatalities revealed that they were more likely to be male (69.6%) and older (Detailed results are provided in Supplementary Table 1 available in Open Science Framework (OSF) repository at the following link: https://osf.io/hqbzr). More than one-half were single (53.6%). Where the information was known, two-thirds (67.0%) of those who died were alone at the time of the fire, 42.0% were known to be smokers, 21.4% had a history of mental health problems, and 30.4% had a history of alcohol dependency. In addition, 14.3% were reported to have mobility issues including use of a walking stick/frame, wheelchair or were ‘otherwise immobile’.

The majority of alcohol-related fatal fires occurred in private residential premises (88.4%). The leading areas of fire origin reported were the sitting/living room (25.0%), and bedroom (23.2%). Likely due to extensive damage because of the fire, the information relating to fire safety equipment was limited but it was known that fire safety equipment was present in 22.3% of the premises of alcohol-related fires. Saturdays (21.4%), and the months of March and December (13.4% respectively) represented more alcohol-related fatal fires.

**3.3.2 Alcohol-related fatal fires versus non-alcohol-related fatal fires**

Supplementary Table 1 compares the characteristics of alcohol-related fatal fires to fatal fire incidents where alcohol was not involved to identify features that may be specific to alcohol-related fatal fires. Although fire fatalities overall were more common among older people, fire fatalities in the 35–49 years age group were significantly more likely to involve alcohol (65.9%) (p<0.001). Alcohol-related fires were more likely among smokers (p=0.001), those with a history of alcohol dependency (p<0.001), and those whose employment status was ‘other’ (p=0.002). Alcohol-related fatal fires were more likely when friends were present at the time of the incident (p<0.001).

**3.3.3 Regression analysis**

Table 3 provides the findings from a logistic regression carried out to assess the effect of the independent variables most strongly associated with alcohol-related fatal fires, with a p-value of <0.05 from Supplementary Table 1. History of alcohol dependency was a significant predictor of a fatal fire being alcohol-related (OR=4.109, 95% CI: 1.118, 15.106, p=0.033).

**Table 3.** Logistic regression of risk factors associated with the likelihood of a fatal fire being alcohol-related.

Characteristics	Odds ratio	95% confidence interval (lower)	95% confidence interval (upper)	p-value
**Age group**				
0–34	REF			
35–49	2.052	0.134	31.326	p=0.605
50–64	1.172	0.085	16.100	p=0.905
65–84	1.468	0.096	22.568	p=0.783
85+	3.851	0.154	96.239	p=0.412
**Smoker**				
No	REF			
Yes	1.183	0.219	6.402	p=0.845
**Employment status**				
Employed/working	REF			
Unemployed	0.965	0.101	9.242	p=0.975
Home duties	0.157	0.006	4.207	p=0.270
Retired/unable to work due to disability	0.253	0.030	2.120	p=0.205
Other	0.158	0.013	1.893	p=0.145
**Company at time of incident**				
Alone	REF			
Friends	0.714	0.049	10.421	p=0.806
Family/partner	0.230	0.035	1.518	p=0.127
Other	0.280	0.034	2.311	p=0.237
**Mobility issues**				
No	REF			
Yes	1.983	0.442	8.885	p=0.371
**History of alcohol dependency**				
No	REF			
Yes	4.109	1.118	15.106	p=0.033

## 4. Discussion

This is the first study in Ireland to examine alcohol and its role in fire fatalities. The results presented here indicate that alcohol is a key risk factor for fatal residential fires. We found a strong positive association between having a history of alcohol dependency and the likelihood of a fatal fire being alcohol-related. This finding should be considered along with other contextual factors to provide a more comprehensive understanding of the risk factors associated with alcohol-related fatal fires such as smoking, mobility issues, older age, being alone and other external risk factors.

Our study found that over three-quarters of alcohol-related fire fatalities were alone at the time of the incident (67.0%), a key risk factor identified in several other studies examining residential fires. ^7, 10, 14, 38^ Being alone at the time of the fire is an obvious risk factor due to the vulnerability of not having immediate assistance to detect, and escape from a fire, risky behaviours may also be more likely when drinking alone as others are not around to advise against or prevent such activities.

Smokers were at a higher risk of alcohol-related fires (54.7%) compared to fires where alcohol was not involved and previous studies have noted the risk of drinking alcohol and falling asleep while smoking as a common cause of residential fires. ^31, 44, 45^ Where known, of the alcohol-related fires, smoking materials and cooking appliances were noted as contributory factors to fire ignition, suggesting that smoking and/or cooking while under the influence of alcohol poses a genuine risk. The practice of cooking while intoxicated is recognised as a risk factor for fire accidents in the home, including fatal injuries. ^11, 46, 47^ Given the notable shift to off-trade alcohol sales, drinking at home has become commonplace and this should be considered in the context of these findings.

There is limited prior evidence on the geographical characteristics of alcohol-related fire fatalities although previous research has identified rural areas as being a risk factor for fatal residential fires in general. ^32, 33, 37, 48^ Our study found that alcohol-related fatal fires in rural areas were over-represented. Those living in rural areas are potentially at a greater risk likely due to the decreased likelihood of a fire being witnessed and subsequently reported, the higher likelihood of older homes that may be exempt from the same building regulations that newbuilds are, and the travel distances from fire services. We found that travel time to the incident from the nearest fire station was significantly longer when the fire occurred in a rural area. Other geographical characteristics associated with fatal fires identified in the literature indicate a higher prevalence in areas of deprivation. ^10, 30, 31, 36, 49– 52^ There are several explanations why those living in areas that are considered more deprived may be at higher risk of residential fires, including poor quality housing, the use of standalone heaters to avoid the expense of heating oil etc., lack of fire safety equipment, or older properties with fewer building regulations compliance. ^12, 50^ However, area of deprivation was not a significant predictor of a fatal fire being alcohol-related in this study, consistent with findings from a US study. ^50^

This study has several strengths. The role of alcohol in fatal fires has not been considered in Ireland to date. Examining these incidents through a geographical lens, using precise location data, allowed a greater insight into the areas at highest risk of alcohol-related fires which may provide a novel approach to informing more focussed fire prevention initiatives. The toxicology reports permit a greater insight into the substances used by the individual before their death, including their level of alcohol use and whether other substances were consumed which may have further increased their risk of harm.

The small sample of alcohol-related fatal fires was a limitation of our analysis. Missing or unknown/not recorded data was also an issue. Due to the nature of fire incidents, damage or obliteration of evidence is commonplace, therefore there were several variables where the information was not obtained, especially in relation to the cause or source of the fire. Furthermore, as many of the fatalities were alone at the time of the incident, witness testimonials were not available to provide further information.

The analysis presented here considered the travel time from the nearest fire station to the location of the fatal fire, however it is not known if this was the fire station that despatched the fire engine to attend the scene or whether one was dispatched at all. Furthermore, travel times presented here did not consider whether routes were congested or restricted. It was also unknown at what stage fire services were alerted to the scene, i.e. the fire may have been well progressed by the time they were notified and therefore travel time may not be relevant. It should also be noted that some fires lead to more than one fatality, and the characteristics of these fires will therefore have greater representation in our data.

Drinking at home may pose additional risks due to larger measures being poured and consumed, as well as drinking for longer periods of time. This in turn may lead to riskier behaviours such as cooking while intoxicated and less care when tending to open fires. The evidence presented here is an important step in ensuring that alcohol use is considered when planning fire safety campaigns and the use data in this study is used to promote policy and practice. Alcohol use alone is not the leading risk factor for fatal fires but when combined with the other risk factors identified, increases the risk of accidental fires and potentially death.

Several recommendations can be proposed based on the findings of this study to help prevent alcohol increasing the risk of fire fatalities. These include provision of specialist alarms (e.g. louder and/or visual alarms, sprinkler systems, etc.) in the homes of those known to be alcohol dependent; targeted prevention campaigns at those living in older homes that precede the building regulations required of newer builds; implementation of rotating advice on labelling on alcohol products to include the risk of fire when cooking while intoxicated; urging family members, neighbours and friends to identify those in their community at greater risk and discourage hoarding, the use of candles, chip pans, and open fires, when drinking; and finally, to include alcohol as a risk factor in annual fire safety week.

Further research is required to deepen our understanding of alcohol-related fatal fires. Due to limited information about the presence of fire safety equipment, there is still more to be learned about their effectiveness in this study and further research to examine their use in non-fatal fires is warranted. Examining in greater detail, non-fatal fire hospitalisations where alcohol had been consumed, may identify protective factors for surviving residential fires.

However, knowing who is at an increased risk, and the geographical characteristics of fatal fires will inform policy development. Furthermore, in publicising the risk associated with alcohol use and fatal fires widely, public awareness will increase, encouraging preventative behaviours as well as potentially reducing hazardous drinking patterns. The findings can also provide advocacy groups with further evidence of the harms associated with alcohol use. This study allows a greater understanding of fatal fires and how alcohol represents a risk factor in such incidents which is rarely included when discussing alcohol-related harms more generally. These findings can also be used to inform alcohol-specific policies such as understanding the impact of the Public Health (Alcohol) Act, 2018, as well as contributing to a health impact assessment, recommended in consideration of proposed legislation to increase alcohol availability, the Intoxicating Liquor Bill, 2024 and the Sale of Alcohol Bill, 2021. ^53– 56^

## 5. Conclusion

Over two in every five of those who died in a fire in Ireland had been drinking prior to the incident indicating that alcohol is a key risk factor for fire accidents and mortality, which is not often recognised. Alcohol-related fire fatalities are more common in rural areas and among those who are alcohol dependent. Fire prevention and safety efforts should seek to mitigate the impact of alcohol on increasing the risk of fatal fires.

## Ethical approval statement

The information in this study is obtained from the National Drug-Related Deaths Index (NDRDI). Ethical approval for the data collection, and subsequent use (including analysis and publications) was received from Health Research Board ethics committee (no longer in operation), formerly located at Health Research Board, 73 Lower Baggot Street, Dublin 2, and from ethics committees covering each individual acute hospital providing hospitalisation data. Any changes to the remit of the NDRDI have been approved by the Irish College of GPs Research Ethics Committee (ICGP), Irish College of GPs, 15 Hogan Place, Dublin 2, D02 DK23. Approval for the use of Central Treatment List data was obtained from the Methadone Prescribing Protocol Implementation Committee. All work was carried out in accordance with International Epidemiological Association/European Epidemiology Group guideline document. ^57^

This study does not have an ethics approval reference number because it was approved prior to the introduction of formal numbering systems. At that time, the ethics committee issued confirmation letters rather than assigning approval codes. The original confirmation letter documenting the committee’s approval is available and can be provided upon request.

As the data used in this study are anonymised mortality data and no direct interaction with participants, informed consent was not possible.

## Data Availability

The data used in this study are not publicly available due to ethical and legal restrictions. The dataset comprises individual-level information on deaths, including potentially identifying variables and highly sensitive personal data relating to health status, cause and circumstances of death. Even where direct identifiers are removed, there remains a risk of re-identification, particularly in rare cases or small populations. Access to the data is therefore restricted under the terms of ethical approval and applicable data protection legislation. The data may only be accessed by authorised researchers for approved purposes and cannot be shared or disseminated in an open repository. Requests for access to aggregated or non-disclosive summary outputs may be considered, subject to disclosure control review and the permissions of the data controller. To apply for access to the NDRDI dataset, requests should be made using the application form available on the HRB website https://www.hrb.ie/data-collections-evidence/about-drug-and-alcohol-deaths/ and sent to NDRDI@hrb.ie for consideration. Open Science Framework (OSF). Geographical characteristics and other factors associated with alcohol-related fatal fires in Ireland 2014 – 2021. https://osf.io/hqbzr. ^58^ This project contains the following extended data:
•
*Supplementary Table 1* (Demographic information of fire fatalities, alcohol-related and non-alcohol-related) •
*Supplementary Table 2* (Mean annual rate of alcohol-related fatal fires per 100,000 of county population, and mean annual rate per 100,000 of urban and rural population of each county of alcohol-related fatal fires, 2014 – 2021 •
*Supplementary Table 3* (Geographical characteristics of alcohol-related fatal fires, 2014 – 2021.) •
*Supplementary Table 4* (Distance and travel time between nearest fire station and location of alcohol-related fatal fires, urban and rural areas.) •
*Supplementary Table 5* (Distance and travel time between nearest fire station and location of alcohol-related fatal fires, by deprivation status.) Data are available under the terms of the Creative Commons Attribution 4.0 International (CC BY 4.0) licence.
