# Supplementary material for: Geographical characteristics and other factors associated with alcohol-related fatal fires in Ireland 2014 – 2021
Source: HRB Open Res. 2026 Apr 16;9:15. Originally published 2026 Feb 10. [Version 2] doi: 10.12688/hrbopenres.14341.2 (PMC13122147; doi:10.12688/hrbopenres.14341.2)
Supplement: Peer review history

# Reviewer report: Reviewer response for version 2

**DOI:** 10.21956/hrbopenres.15900.r54719

**Published:** 2026-04-27

**Authors:** James M. Clay, Keegan W. Lawrence

**Affiliations:** 1 Department of Community Health and Epidemiology, Dalhousie University (Ringgold ID: 152977), Halifax, Nova Scotia, Canada; 2 Canadian Institute for Substance Use Research (CISUR), University of Victoria, Victoria, British Columbia, Canada

The authors have adequately addressed the concerns raised in the initial review and we are happy to change the status to Approved. Congratulations on an interesting piece.

 Keegan W. Lawrence, MSc & James M. Clay, PhD

Is the work clearly and accurately presented and does it cite the current literature?

Yes

If applicable, is the statistical analysis and its interpretation appropriate?

Partly

Are all the source data underlying the results available to ensure full reproducibility?

No

Is the study design appropriate and is the work technically sound?

Partly

Are the conclusions drawn adequately supported by the results?

Yes

Are sufficient details of methods and analysis provided to allow replication by others?

Partly

Reviewer Expertise:

Alcohol epidemiology, alcohol and health, alcohol policy, and the determinants of alcohol use.

We confirm that we have read this submission and believe that we have an appropriate level of expertise to confirm that it is of an acceptable scientific standard.

# Reviewer report: Reviewer response for version 1

**DOI:** 10.21956/hrbopenres.15793.r53656

**Published:** 2026-03-19

**Authors:** Michael Ronan Lucey

**Affiliations:** 1 University of Wisconsin School of Medicine and Public Health, Madison, USA

Thank you for the opportunity to review this fascinating study.  As an investigator into alcohol-related harms, particularly the nexus of alcohol use and liver injury, I was aware of many of the other negative consequences on health of excessive alcohol use including motor vehicle accidents, accidents at work and domestic violence.  I had not come across death due domestic fires before. Following reading Ms. Doyles paper, I will add it to the list.

 Ms. Doyle and her co-authors appropriately to demonstrate the importance of local social infrastructure and culture to their analysis of the data under review.  While the number of fatalities is not high, the conclusions are plausible.   A considerable proportion of Irish persons dying in a domestic fire have a positive blood alcohol, and many have high levels.  Persons with a history of alcohol use disorder, who are living (and presumably drinking) alone and/or living in rural areas are at greatest risk. Of these issues, only a history of AUD was independently associated with mortality, however.  Interestingly, the degree of social deprivation was not associated with mortality and alcohol use.

 My only suggestion to the authors would be to include a comment on the non-fatal outcome of many fires. Extrapolating from their data, it would be interesting to capture the morbidity associated with domestic fires in Ireland in the same period.  I would hypothesize that alcohol consumption was also associated in fires which resulted in at least one involved person required admission to hospital.  Such an expansion of the dataset would provide further context about the social and personal toll of drinking in relation to domestic fires.

Is the work clearly and accurately presented and does it cite the current literature?

Yes

If applicable, is the statistical analysis and its interpretation appropriate?

Yes

Are all the source data underlying the results available to ensure full reproducibility?

Yes

Is the study design appropriate and is the work technically sound?

Yes

Are the conclusions drawn adequately supported by the results?

Yes

Are sufficient details of methods and analysis provided to allow replication by others?

Yes

Reviewer Expertise:

I am a clinical hepatologist with an interest in alcohol-associated liver disease and liver transplantation.  I am funded by the NIH to participate in multi center studies of selection for and outcome of liver transplantation in persons with liver failure and short intervals of sobriety.

I confirm that I have read this submission and believe that I have an appropriate level of expertise to confirm that it is of an acceptable scientific standard.

# Reviewer report: Reviewer response for version 1

**DOI:** 10.21956/hrbopenres.15793.r53660

**Published:** 2026-03-09

**Authors:** James M. Clay, Keegan W. Lawrence

**Affiliations:** 1 Department of Community Health and Epidemiology, Dalhousie University (Ringgold ID: 152977), Halifax, Nova Scotia, Canada; 2 Canadian Institute for Substance Use Research (CISUR), University of Victoria, Victoria, British Columbia, Canada

**Summary**

 This paper examines alcohol involvement in fatal fires in Ireland between 2014 and 2021 using coronial data from the National Drug-Related Deaths Index. The authors present descriptive analyses of toxicological findings, a geospatial analysis of fire locations by urban-rural classification, deprivation status, and proximity to fire stations, and a multivariable logistic regression identifying predictors of a fatal fire being alcohol-related. This is a valuable and timely contribution to an underexplored area; to our knowledge it is the first study of its kind in Ireland. The use of detailed coronial data with individual-level toxicology is a particular strength, and the findings have clear relevance for fire prevention policy. The comments below are intended to support the authors in strengthening methodological transparency and the precision of a small number of interpretive claims, and we hope they are taken in that spirit.

**Presentation and literature**

 The manuscript is clearly written and well referenced. One minor suggestion: the abstract and discussion describe history of alcohol dependency as "the only significant risk factor" in the regression. Given the wide confidence intervals for other variables in Table 3, which reflect the modest sample size rather than a meaningful absence of association, it may be worth adding a brief qualifier to acknowledge that limited statistical power may have precluded detection of effects for other variables. This would help readers avoid interpreting non-significance as evidence of no association.

**Technical soundness**

*Definition of alcohol-related.* Table 1 indicates that the alcohol-related category includes cases with BAC recorded as "present/trace (<10 mg/100 mL)." It would be helpful to briefly justify this inclusive definition, as the contribution of trace alcohol to fire risk in these cases is uncertain. A short sensitivity analysis using a higher threshold (e.g. ≥50 mg/100 mL) would reassure readers that the main findings, including the headline 41% prevalence, are robust to this definitional choice.

*Statistical independence.* The paper notes that some fires involved more than one fatality. It would be helpful to report the number of unique fire incidents alongside the number of individual fatalities. The authors may also wish to consider a sensitivity analysis excluding multi-fatality incidents, or at minimum acknowledge that clustering within incidents may affect the independence assumption of the regression model.

*Variable selection for regression.* The decision to restrict the multivariable model to variables significant at p<0.05 in bivariate analyses is understandable given the sample size, and the authors acknowledge this as a limitation. It may be worth noting specifically whether any variables with strong theoretical relevance (such history of mental health problems) were excluded on this basis, so readers can assess the potential impact on the findings.

**Reproducibility**

 The geospatial workflow is described as mapping Eircodes "using Google Maps." A brief additional description of how the nearest fire station was identified and what routing settings were used for distance and travel time would make this analysis more readily replicable by others.

 The appendix helpfully provides "not known/not recorded" categories for several variables. It would further strengthen the paper to include a short summary of missingness across key variables, and to state the analytic sample size for the each analysis explicitly. Given the extent of missingness and cell suppression across some variables, it is currently difficult to determine how many cases were included in the multivariable model.

**Statistical interpretation**

 Three points on interpretation for the authors to consider. First, and most importantly, Supplementary Table 1 shows that the urban-rural distribution does not differ significantly between alcohol-related and non-alcohol-related fatal fires ( *p*=0.703). This makes it difficult to sustain the claim that rural over-representation is a finding specific to alcohol involvement, and we would encourage the authors to revisit the framing of this result in both the results and discussion sections. It may be more accurate to characterise the rural pattern as a feature of fatal fires generally in Ireland, with alcohol-related fires reflecting that broader distribution rather than showing a distinct geographical signal.

 Second, the finding that no alcohol-related fatal fires occurred in affluent areas is noteworthy, but should be interpreted cautiously. With relatively few cases overall and cell suppression limiting what can be observed in Supplementary Table 3, it is difficult to determine whether the apparent zero in affluent areas reflects a genuine absence of alcohol-related fire risk in those populations, or simply the general scarcity of fatal fires in more affluent areas; which may itself reflect factors such as housing quality or fire safety equipment rather than anything specific to alcohol. A brief note acknowledging these interpretive constraints would be a helpful addition for readers.

 Third, Figure 1 shows that 48.7% of all fatal fires occurred in rural areas, which is very similar to the 47.3% figure for alcohol-related fires specifically, consistent with the non-significant bivariate result noted above.

**Minor points**

 The paper would benefit from a brief comment on whether the COVID-19 years (2020–2021) show any distinct pattern, given the well-documented shifts in drinking behaviour and home time during that period that the authors themselves reference in the introduction.

 Overall, this paper meaningfully contributes to understanding alcohol involvement in fatal fires in Ireland and is valuable for harm prevention planning. Some minor clarifications and sensitivity checks would enhance the transparency of the methods, strengthen confidence in the findings, and improve interpretability for policy and practice audiences.

 Keegan W. Lawrence & MSc & James M. Clay, PhD

Is the work clearly and accurately presented and does it cite the current literature?

Yes

If applicable, is the statistical analysis and its interpretation appropriate?

Partly

Are all the source data underlying the results available to ensure full reproducibility?

No

Is the study design appropriate and is the work technically sound?

Partly

Are the conclusions drawn adequately supported by the results?

Yes

Are sufficient details of methods and analysis provided to allow replication by others?

Partly

Reviewer Expertise:

Alcohol epidemiology, alcohol and health, alcohol policy, and the determinants of alcohol use.

We confirm that we have read this submission and believe that we have an appropriate level of expertise to confirm that it is of an acceptable scientific standard, however we have significant reservations, as outlined above.
